# PD192 Criteria For Decision-Making In Health Economic Evaluations: An Analysis Of Global Practices

**DOI:** 10.1017/S0266462324004148

**Published:** 2025-01-07

**Authors:** Celina Borges Migliavaca, Verônica Colpani, Miriam Allein Zago Marcolino, Maicon Falavigna, Carisi Anne Polanczyk

## Abstract

**Introduction:**

Economic criteria have become critical for health technology assessment (HTA) due to the rising costs of health technologies. The use of explicit, predefined criteria for economic evaluations enhances the transparency, objectivity, and predictability of the process. We aimed to identify which organizations worldwide integrate explicit and predefined criteria into their health economic analyses during HTA.

**Methods:**

We conducted a scoping review to identify organizations responsible for HTA processes at the national level in any country. The identification of eligible organizations was carried out by reviewing members of the EUnetHTA, HTAsiaLink, INAHTA, and RedETSA networks as well as organizations evaluated in reviews with a similar scope. For each eligible organization, information was extracted on the inclusion of economic factors during decision-making and the existence of predefined criteria for judging the results of economic evaluations.

**Results:**

Sixty-nine organizations from 56 countries were identified, of which 66 (96%) considered economic factors for HTA. Fifty-two (79%) organizations conducted cost-effectiveness analyses, 42 (64%) assessed budget impact, and one focused solely on total technology costs. Thirty-four organizations (51%) declare not having criteria for economic evaluation, whereas 14 (21%) from 12 countries had explicit criteria. There were no data found for 18 organizations (27%). Among the organizations with explicit criteria, 11 (17%) applied willingness-to-pay thresholds in cost-effectiveness evaluations and five (8%) applied criteria related to budget impact for decision-making, such as a maximum percentage of budget impact.

**Conclusions:**

Although most organizations consider economic factors for HTA, many do not have explicit, predefined criteria for decision-making. Among those that presented such criteria, willingness-to-pay thresholds for cost-effectiveness analyses were the most common. The findings of this study also help to identify complementary factors that can be considered to promote greater systematization and transparency in the decision-making process.

